# Increased ^18^F-FDG Uptake in the Obturator Muscles Due to Denervation-Driven Atrophy: Teaching point: Denervated muscles can show a temporary increase in ^18^F-FDG uptake on PET-CT, thus simulating malignancy

**DOI:** 10.5334/jbsr.1832

**Published:** 2019-05-16

**Authors:** Thomas Panis, Lode Goethals, Frank De Geeter

**Affiliations:** 1 UZB, BE; 2 AZ Sint-Jan, BE

**Keywords:** ^18^F-FDG, denervation, atrophy, oncology, muscle

## Case Report

**Figure 1:**
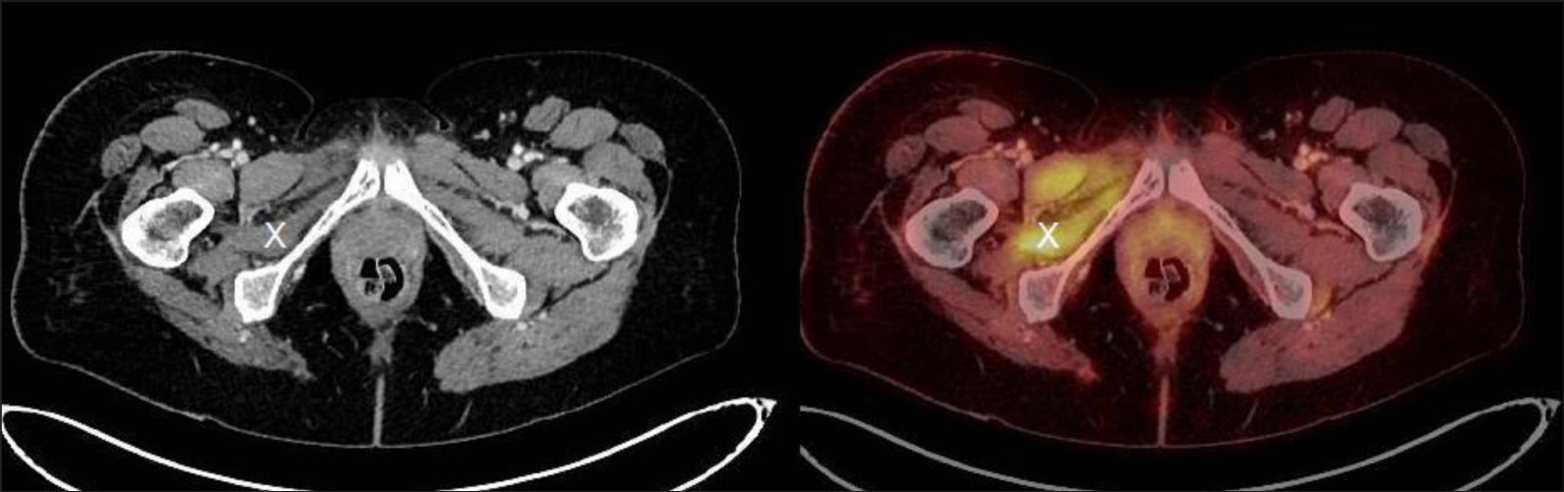


**Figure 2:**
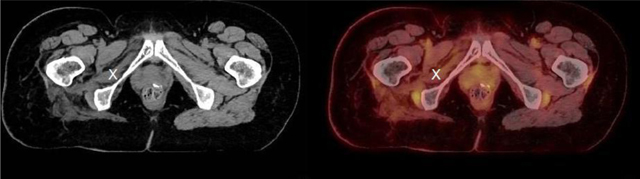


A 52-year-old female patient with a history of rectal carcinoma underwent targeted radiotherapy on right-sided pelvic lymph node metastases (42 Gy in 21 fractions). She later developed local post-radiation retroperitoneal fibrosis with resulting ureteric stenosis. Serial oncological follow ups with ^18^F-FDG PET-CT showed a right-sided increased uptake in the obturator internus (Figures 1 and 2, X) and obturator externus muscles, at a significant distance inferiorly to the irradiated zone. This uptake first became visible 22 months after the last radiotherapy session, reached a peak after 25 months (Figure 1), and all but disappeared after 32 months without any further oncologic treatment. It was associated with gradual and permanent muscular volume loss compared to the contralateral side. This volume loss reached a maximum after 37 months (Figure 2). The previous pelvic irradiation suggests that post-irradiation scarring led to injury of the nerves innervating the obturator muscles (i.e., the lumbar plexus or the obturator nerve).

## Comment

Temporary glucose hypermetabolism in denervated muscle has been described in small patient groups. The exact mechanism of this hypermetabolism has not yet been elucidated. One proposed mechanism is the immunohistochemically proven increase of certain cellular membrane proteins (glucose transporter type 1, acetylcholine receptors) in response to denervation. Another theory links the rise in metabolism to widespread apoptosis – an energy-consuming process. Hypermetabolism due to denervation can be important in an oncological context, because unexpected FDG uptake increase in regional skeletal muscles might lead to false-positive diagnoses of cancer recurrence or metastases [1].
